# A Tobacco-Derived Thymosin *β*4 Concatemer Promotes Cell Proliferation and Wound Healing in Mice

**DOI:** 10.1155/2016/1973413

**Published:** 2016-07-14

**Authors:** Rylosona Janarthini, Xiaolei Wang, Lulu Chen, Lei Gao, Lingxia Zhao

**Affiliations:** ^1^Joint Tomato Research Institute, School of Agriculture and Biology, Shanghai Jiao Tong University, Shanghai 200240, China; ^2^Plant Biotechnology Research Center, School of Agriculture and Biology, Shanghai Jiao Tong University, Shanghai 200240, China

## Abstract

Thymosin *β*4 (T*β*4) is a peptide that is known to play important roles in protection, regeneration, and remodeling of injured tissues in humans, and that shows great promise in a range of clinical applications. However, current strategies to T*β*4 are insufficient to meet growing demand and have a number of limitations. In this current study we investigated whether expression of recombinant T*β*4 in plants, specifically in tobacco (*Nicotiana tabacum*) leaves, represents an effective approach. To address this question, a 168 bp* Tβ4* gene optimized for tobacco codon usage bias was constitutively expressed in tobacco as a 4-unit repeat concatemer, fused to a polyhistidine tag. Quantitative polymerase chain reaction and Western blot analyses were used to verify* 4×Tβ4* expression in 14 transgenic tobacco lines and enzyme-linked immunosorbent assay analysis indicated 4×T*β*4 protein concentrations as high as 3 *μ*g/g of fresh weight in the leaves. We observed that direct administration of tobacco-derived T*β*4 was more effective than T*β*4 either obtained commercially or derived from expression in* Escherichia coli* at promoting splenocyte proliferation* in vitro* and wound healing in mice through an endothelial migration assay. This study provides new insights into the development of plant-derived therapeutic proteins and their application by direct administration.

## 1. Introduction

Thymosin *β*4 (T*β*4), a 43-amino acid peptide that is encoded by the* TMSB4X* gene on the X chromosome of mice and humans, was initially isolated from thymosin fraction 5 (TF5) as a biologically active component [1]. TF5 was originally authorized by the FDA (Food and Drug Administration) to treat the primary immunodeficiency disease, DiGeorge syndrome, in clinical trials involving young children in the US [2]. T*β*4 was initially identified as an actin monomer (G-actin) binding protein and has the capacity to sequester G-actin, thereby inhibiting intracellular actin polymerization [3–5]. In addition to regulating actin formation, extracellular T*β*4 participates in several biological processes, including blood coagulation, osteoblast differentiation, activation and degranulation of platelets, and regulation of cell migration (http://www.ncbi.nlm.nih.gov/gene/7114). T*β*4 therefore plays an important role in medical treatments, such as anti-inflammation [6], angiogenesis [7, 8], remodeling of damaged tissues [9, 10], and the prevention of organic fibrogenesis [5, 11, 12].

Several studies have demonstrated that the expression of* Tβ4* is upregulated in injured tissue and differentiating cells [13, 14], and high concentrations of T*β*4 protein have been found in blood platelets, wound fluid, and a range of tissues [15, 16] and its effects appear to be widespread. It has been extensively employed to treat diabetic ulcers and bedsores [17, 18] and damaged corneas [10, 19, 20] and for cardiac cell survival and the repair of heart muscle injured during heart attacks [9, 21, 22], as well as antifibrogenesis of the liver [5], kidney [11, 23] and lung [12]. T*β*4 therefore shows great promise for numerous clinical applications [17] and the resulting increased commercial demand relies on efficient production methods. Traditionally, T*β*4 is obtained from extracts of animal thymus glands or is chemically synthesized; however, genetic engineering approaches, such as expression in* Escherichia coli*, have also been developed [24, 25]. It has also been reported that a T*β*4 protein concatemer expressed in* E. coli* is able to promote wound healing in mice [24]. However, for clinical applications,* E. coli* derived T*β*4 protein must undergo a complex purification process to eliminate impurities and endotoxins, which represents a significant disadvantage of this expression system. Overall, the disadvantages to the aforementioned methods include high production costs associated with separation and purification of the target protein, as well as the risk of zoonosis [26, 27].

Current T*β*4 production capacity cannot meet the clinical demand, and plant-based protein/peptide expression systems represent a potentially attractive solution as they are relatively inexpensive, do not have problems associated with zoonosis, and are scalable. Accordingly, over the last decade plant-based expression systems have been extensively used to synthesize therapeutic proteins, antibodies, and vaccines [26–28]. In order to meet the rising clinical demand for T*β*4, we wanted to develop a novel expression system to produce T*β*4 protein at a lower cost, in a safe manner, and through a process that is readily scalable. To this end, a T*β*4 protein encoding gene (*Tβ4*) was designed to take into account tobacco (*Nicotiana tabacum*) codon usage bias and was integrated into the tobacco genome via* Agrobacterium*-mediated transformation. We describe here the production of tobacco-derived 4×T*β*4 protein and report that it can promote cell proliferation* in vitro* and wound healing* in vivo* in mice. Furthermore, we present evidence that the plant-derived T*β*4 protein, which can be directly applied to wounds, is more bioactive than the heterologous form derived from overexpression in* E. coli*. The study provides important information for the future development of plant expressed pharmaceutical proteins for direct administration.

## 2. Materials and Methods

### 2.1. Biological Materials

Tobacco (*N. tabacum *cv. Bairihong) seeds and DH5*α* (*E. coli*) and EHA105 (*A. tumefaciens*) cells were obtained from the Plant Biotechnology Research Centre, School of Agriculture and Biology, Shanghai Jiao Tong University, China. Six- to eight-week-old, healthy Balb/c mice were purchased from the Animal Centre, Shanghai Jiao Tong University, Shanghai, China.

### 2.2. Construction of the Plant Expression Vector

Based on the T*β*4 amino acid sequence [1], a 168 bp* Tβ4* gene optimized for tobacco codon usage bias was designed, synthesized, and subcloned into the pUC57 plasmid. The resulting pUC57-*Tβ4* plasmid was digested with two combinations of restriction endonucleases (*Spe* I/*Sac* I and* Xba* I/*Sac* I) and the released DNA fragment, with* Xba *I/*Sac *I ends, was ligated into the* Spe*I/*Sac*I sites of pUC57-*Tβ4* based on the isocaudameric properties of* Spe *I and* Xba* I to create the pUC57-*2×Tβ4* plasmid. The pUC57-*4×Tβ4 *plasmid with four repeats of the* Tβ4* gene was generated using a similar approach. A DNA sequence encoding six histidines with a* Bam* HI restriction site was introduced into the 5′ end of the* 4×Tβ4 *construct via polymerase chain reaction (PCR) using the PCR primers* Tβ4 *F1: 5′-ggggatccatgcaccaccaccaccaccacggtaccatgtctagaatgtctga-3′ and* Tβ4* R1: 5′-ccgagctcttaact agtcataga-3′. The fused DNA fragment of 6*×his-4×Tβ4* was then digested by* Bam*HI and* Sac*I and subcloned into the 35S::*hIXF* plasmid constructed by the Plant Biotechnology Research Center, Shanghai Jiao Tong University, China, to create the plant binary expression vector, 35S::*6×his-4×Tβ4*. This plasmid was then introduced into EHA105 (*A. tumefaciens*) via the freeze thaw method [29].

### 2.3. Tobacco Transformation and PCR Analysis

Tobacco transformation and extraction of tobacco genomic DNA were conducted as previously described [30], with slight modifications. The concentration of genomic DNA extracted from the regenerated tobacco lines was adjusted to 50 ng/*μ*L for PCR analysis, and the 35S::*6×his-4×Tβ4 *plasmid and genomic DNA derived from untransformed tobacco were used as positive and negative controls, respectively. PCR reactions were performed in 25 *μ*L reaction volumes containing the following components: 2 *μ*L genomic DNA template, 2.5 *μ*L 10x PCR buffer with MgCl_2_, 1.5 *μ*L 2.5 mM/L dNTPs, 1 *μ*L 10 *μ*M each PCR primer (P35S: 5′-ttcgtcaacatggtggagca-3′ and Noster: 5′-aagaccggcaacaggattca-3′), 0.2 *μ*L Ex*Taq* DNA polymerase (5 unit/*μ*L) (Takara Biotechnology, Dalian, China), and 16.8 *μ*L deionized ddH_2_O. The PCR program was one cycle 94°C for 3 min, followed by 35 cycles (94°C for 30 s, 54°C for 30 s, and 72°C for 1.5 min), and finally 72°C for 8 min before being held at 10°C. The PCR products were electrophoresed on a 1.0% (w/v) agarose gel containing 0.5 mg/L ethidium bromide (EB) in 1x TAE buffer (Tris base acetic acid EDTA). Gels were imaged under UV light.

### 2.4. qRT-PCR

Total RNA was extracted from young leaves of both transgenic and nontransgenic tobacco lines using an RNAprep pure plant kit (TIANGEN, Beijing, China) according to the manufacturer's instructions. The total RNA samples were treated with DNase (RNase-free, TaKaRa, Dalian, China) to eliminate DNA contamination. One *μ*g total RNA was used as a template to synthesize complementary DNA (cDNA) using the Prime-ScriptTM RT Master Mix Kit (TaKaRa, Dalian, China) at 37°C for 15 min and 85°C for 5 sec. Two *μ*L cDNA, diluted 100-fold, was used for qRT-PCR with gene specific primers (*Tβ4*F: 3′-acggtaccatgtctagaatg-5′;* Tβ4*R: 3′-ccgagctcttaactagtcatg-5′). The qRT-PCR program was initiated at 95°C for 30 sec, followed by 27 cycles of 95°C for 15 sec, 58°C for 15 sec, 72°C for 25 sec, and finally 72°C for 5 min, before being held at 4°C. The* UBIQUITIN* gene (GenBank: X58253.1) was used as a reference gene for data normalization, using the gene-specific primers UbiF: 3′-aagacctacaccaagcccaa-5′ and UbiR: 3′-aagtgagcccacacttacca-5′.

### 2.5. Western Blot and ELISA (Enzyme-Linked Immunosorbent Assay) Analyses

Total soluble protein (TSP) was extracted from young leaves of transgenic and nontransgenic tobacco lines (three biological replicates) using phosphate-buffered saline (PBS) extraction buffer (20 mM sodium phosphate pH 7.4, 137 mM sodium chloride, and 2.7 mM potassium chloride). Approximately 500 mg of each leaf sample was ground to a fine powder using a pestle and mortar with liquid nitrogen and subsequently added to PBS extraction buffer in a 1 : 1 ratio (w/v). The powder and extraction buffer were mixed well using a Vortex-Genie 2 (Scientific Industries, USA) and then transferred to an ice bath for 2 hrs. The extraction mixture was then centrifuged at 12,000 g for 20 min at 4°C and the supernatant collected. The TSP concentration was determined using the Bradford method [31], using bovine serum albumin (BSA) to create a standard curve.

The TSP concentration was adjusted to 0.5 *μ*g/*μ*L with PBS buffer. After boiling for 5 min, 10 *μ*L of the adjusted TSP was mixed with loading buffer (120 mM Tris-HCl pH 6.8, 20% glycerol, 4% SDS, 3%  *β*-mercaptoethanol, and 0.02% bromophenol blue) in equal volumes (v : v = 1 : 1) and separated via sodium dodecyl sulphate/polyacrylamide gel electrophoresis (SDS-PAGE). Subsequently, electrophoretically separated proteins were transferred to a polyvinylidene fluoride (PVDF) membrane (filter pore size 0.22 *μ*m, Bio-Red, USA) for Western blot analysis [24]. The target protein was detected via incubation of the PVDF membrane with a mouse anti-His-tag monoclonal primary antibody (1 : 5,000 dilutions) purchased from Generon Ltd. (Maidenhead, The United Kingdom) and an alkaline phosphatase- (AP-) conjugated goat anti-mouse IgG secondary antibody (1 : 2,000 dilution) (Shanghai ImmunoGen Biological Technology, Shanghai, China). The immunoreactive proteins were visualized by BCIP/NBT (5-bromo-4-chloroindol-3-yl phosphate/nitro blue tetrazolium) staining [30].

ELISA assays were carried out as previously described [32]. The 4×T*β*4 protein derived from* E. coli* was used as a positive control and diluted to concentrations of 4, 2, 1, 0.5, 0.25, 0.125, 0.0625, and 0.03125 ng/*μ*L in PBS buffer [24]. The 50 *μ*L serial dilutions of the* E. coli*-derived 4×T*β*4 and the 4×T*β*4 containing TSP extracted from transgenic tobacco (adjusted to 1 *μ*g/*μ*L) were added to the wells of a 96-well plate in triplicate, and the TSP from nontransgenic tobacco leaves was used as a negative control. The same primary and secondary antibodies were used as for the Western blot analysis. BCIP/NBT was used as substrate for the color reaction and* A*_410_ was measured with a microtiter plate reader (BioTek Instruments, Winooski, VT, USA). 4×T*β*4 protein concentrations were calculated using the previously established standard curve.

### 2.6. Cell Proliferation Assay (*In Vitro*)

Chemically synthesized standard T*β*4 protein was diluted to 10 ng/*μ*L and employed as a positive control. The 4×T*β*4 containing TSP extracted from the young transgenic tobacco leaves was filtered through a 0.45 *μ*m pore size membrane (Millipore Millex, Shanghai Jinxin Bio. Shanghai, China) and the 4×T*β*4 protein concentration adjusted to 10 ng/*μ*L with PBS extraction buffer. TSP (amount equal to the tested sample) derived from nontransgenic tobacco leaves were used as negative control.

The* in vitro* bioactivity of T*β*4 was determined using a 3-(4,5-dimethylthiazol-2-yl)-2,5-diphenyl-2H-tetrazolium bromide (MTT) assay [33]. Spleen cells isolated from 6–8-week-old Balb/c mice were collected via centrifugation at 1,000 ×g for 10 min at room temperature [24]. Pellets of spleen cells were subsequently resuspended and diluted to 1 × 10^5^ cells/mL in RPMI 1640 medium (Sigma-Aldrich, Shanghai, China). A 100 *μ*L spleen cell aliquot was added to each well of a 96-well plate and then 100 *μ*L of the diluted standard T*β*4 protein (1 *μ*g), and the TSP extracted from the transgenic and nontransgenic tobacco leaves and a PBS vehicle control were added separately to triplicate wells. The 96-well plate was incubated at 37°C, 5% (v/v) CO_2_, for 24 h in a cell-culture incubator, and then 10 *μ*L of MTT reagent purchased from Shanghai Hushi Medical Technology Co., Ltd. (Shanghai, China) was added to each well and incubated for an additional 4 h. The plate was periodically observed using a CKX31 inverted microscope (Olympus, Watford, Herts., U.K.) and 150 *μ*L of dimethyl sulphoxide (DMSO) was added to each well when purple precipitate was observed. After swirling gently, the plate was kept in the dark at room temperature for 15 min. Subsequently, the absorbance of each reaction at wavelength of 570 nm was measured with a microtiter plate reader (BioTek, USA).

Spleen cell proliferation was calculated using the following equation:

(1) $$\begin{array}{c} \text{Proliferation}\left(\;\%\;\right)=\frac{\left({\text{D}}_{\text{trial}}-{\text{D}}_{\text{control}}\right)}{{\text{D}}_{\text{trial}}}\times 100. \end{array}$$

### 2.7. Wound Healing Experiment (*In Vivo*)

The 4×T*β*4 containing TSP extracted from young transgenic tobacco leaves were filtered through a 0.45 *μ*m pore size membrane (Millipore Millex, Merck KGaA, Darmstadt, Germany) and freeze-dried (Thermo Fisher Scientific ISS110, USA), before being diluted to 100 ng 4×T*β*4/*μ*L and 50 ng 4×T*β*4/*μ*L with PBS extraction buffer. Nontransgenic tobacco-derived TSP were used as a negative control.

Three, full-thickness, 5 mm punch wounds were inflicted on the dorsal surfaces of each 6–8-week-old Balb/c mouse as previously described [13]. Punch wounds were made on eighteen Balb/c mice and twelve healthy mice were chosen for the experiments. Fifty *μ*L samples of six treatments were then applied at 24 and 48 h after wounding. The six treatments included tobacco-derived 4×T*β*4 proteins (5 *μ*g and 2.5 *μ*g), T*β*4 protein (Purchased from GL Biochem, 5 *μ*g),* E. coli*-derived 4×T*β*4 protein (5 *μ*g), nontransgenic tobacco TSP (5 *μ*g and 2.5 *μ*g), and PBS as a vehicle control. Three biological replicates were carried out for each experimental treatment.

From days 2 to 10 after treatment, keratinocyte migration from six mice was examined by measuring the distance between epidermal tongues of the wound edges with a Vernier caliper (Endura-Greenlee Tools, E0531, Shanghai, China). Wound closure was calculated using the formula described by Li et al. (2007) [25]:

(2) Wound closure%=distance of migrated keratinocytes from the wound edgetotal wound width×100.

To examine reepithelialization and vessel counts of the wound, six additional mice (treated as above) were euthanized on day 8 after treatment and tissues from the healing wounds collected and fixed in 4% (v/v) formalin buffer. The fixed tissues were then embedded in paraffin after dehydration in a series of ethanol concentrations (75%, 85%, 95%, and 100%) and 5 *μ*m sections from the middle of the wounds were made using a microtome (Leica, RM2200) [24]. Sections were then mounted on glass slides and stained with hematoxylin and eosin (Shanghai Dingjie Biotechnology Company, Shanghai, China) after paraffin removal. Vessel counts in the wound beds were determined by identifying vascular spaces distinguished by their endothelial lining, including those at the junction of the dermis and the hypodermis, as angiogenesis within wounds occurs to a great extent from these vessels. Counts were averaged as vessel counts per 10 high-powered fields (40x).

## 3. Results

### 3.1. Design, Transformation, and Molecular Examination of the T*β*4 Transgene

**Figure 1:**
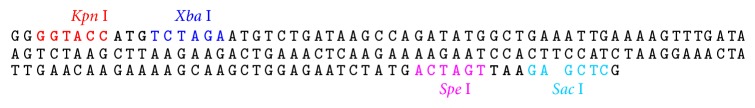


Based on the T*β*4 amino acid sequence (Genpept accession, P62326.2), an 168 bp* Tβ4* gene optimized for tobacco codon usage bias was designed and synthesized, including the restriction endonuclease sites (colored below)* Kpn *I/*Xba *I and* Spe *I/*Sac *I at the 5′ and 3′ ends, respectively, protection bases, and both an initiation codon (**ATG**) and a termination codon (**TAA**) (bold). The sequence was as shown in Figure 1.

**Figure 2:**
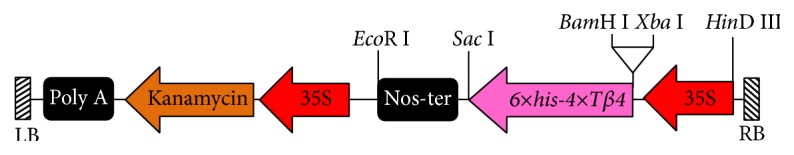
Diagram of the plant binary vector, 35S::*6×his-4×Tβ4*. LB: T-DNA border (left), poly A: CaMV 35S poly A, 35S: CaMV 35S gene promoter, Kanamycin: CDS (coding sequence) of* NPTII* (neomycin phosphotransferase II) gene, Nos-ter:* NOS *(nopaline synthetase) gene terminator,* 6×his-4×Tβ4*: the* 4×Tβ4 *gene with DNA sequence encoding* 6×his *at the 5′ end, and RB: T-DNA border (right).

*4×Tβ4 *and* 35S*::*6×his-4×Tβ4 *constructs were subsequently created (Figure 2) and transformed into tobacco and the resulting putative transgenic tobacco lines were screened by PCR. This revealed an 1,140 bp DNA band, corresponding to the 4*×Tβ4* gene, in the positive control and in 14 of the putative tobacco lines, while this DNA band was not present in nontransformed tobacco samples (Supplementary Appendices 1-d to f and Appendix 2 in Supplementary Material available online at http://dx.doi.org/10.1155/2016/1973413). We concluded that the* 4×Tβ4 *gene had been successfully integrated into the tobacco genome of the positive lines.

### 3.2. Expression of the 4×T*β*4 Gene

**Figure 3:**
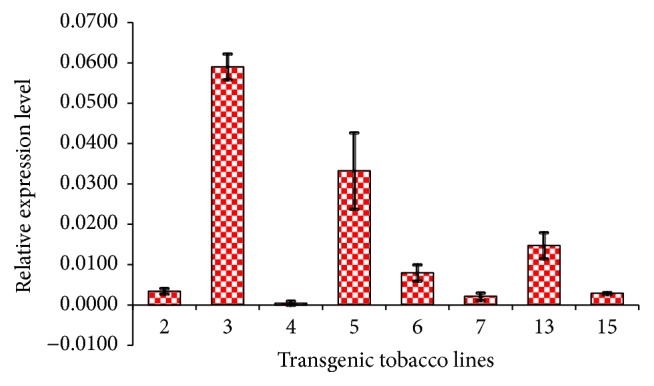
The differences in relative expression levels between the transgenic tobacco lines. Bars represent the expression levels of different transformed tobacco lines (*n* = 3).

Expression of the* 4×Tβ4 *gene was evaluated in eight of the positive transgenic tobacco lines by qRT-PCR. The expression levels varied substantially, between lines, with lines 3, 5, and 13 having high levels of* 4×Tβ4* transcript accumulation, and line 3 showing particularly high expression. The expression level in other lines ranged from relatively low (lines 2, 6, 7, and 15) to nondetectable (line 4) (Figure 3). These differences may be attributed to the copy number of the integrated* 4×Tβ4 *gene, as well as their sites of integration in the tobacco genome [34, 35].

### 3.3. Verification of Successful Expression of the Recombinant 4×T*β*4 Protein in Transgenic Tobacco

The TSP extracted from young leaves of both transgenic and nontransgenic tobacco (negative control) was examined by Western blot and ELISA analyses using anti-His-tag monoclonal primary antibody. The TSP content derived from young tobacco leaves ranged from 4.92 mg/g fresh weight (FW) (line 2) to 6.18 mg/g FW (line 13). Western blot analysis showed a 23.2 kDa band in transgenic tobacco lines, which corresponded to the predicted size of the recombinant protein and which was absent from the nontransgenic tobacco extracts (Supplementary Appendix 4).

The 4×T*β*4 protein content in the transgenic young tobacco leaves was determined by ELISA. The concentrations of 4×T*β*4 protein in transgenic tobacco extracts ranged from 0.492 *μ*g/g FW in line 6 to 2.946 *μ*g/g FW in line 5 (Supplementary Appendix 3). Differences in the expression level of the 4×T*β*4 recombinant protein between the lines may be attributed to the number of the* 4×Tβ4* copies and position of integration, as well as to posttranscriptional processes [36, 37].

### 3.4. Tobacco-Derived 4×T*β*4 Protein Promotes Cell Proliferation (*In Vitro*)

**Figure 4:**
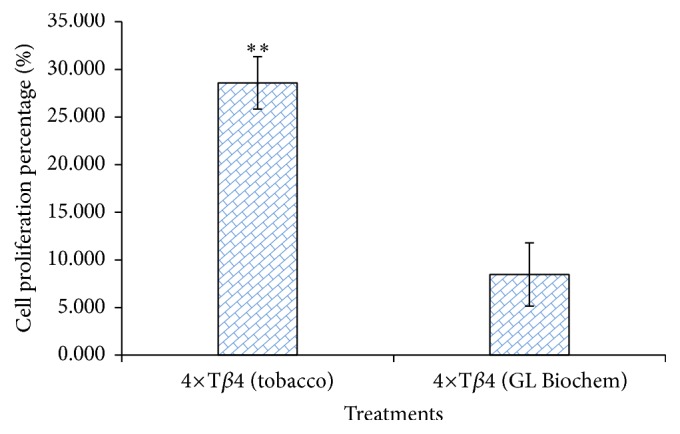
Assay of tobacco-dervied 4×T*β*4 protein promoting mice splenic lymphocyte proliferation using the 3-(4,5-dimethylthiazol-2-yl)-2,5-diphenyl-2H-tetrazolium bromide (MTT) method. Column* 4×Tβ4 *(tobacco) indicates* 4×Tβ4* extracted from transgenic tobacco leaves; column* Tβ4* (GL Biochem) indicates commercially purchased* Tβ4*; *∗∗* indicates that tobacco-derived* 4×Tβ4* was significantly more effective than commercial* Tβ4* at promoting cell proliferation (*n* = 6, *p* < 0.01).

A mouse splenic lymphocyte proliferation assay (MTT) was used to determine the biological activity of the tobacco-derived 4×T*β*4 protein. Application of the 4×T*β*4 protein (1 *μ*g) derived from transgenic tobacco leaves (line 5) caused a 28.59 ± 4.97% increase in splenic lymphocyte proliferation, which was significantly higher than the effects of applying either 1 *μ*g of the commercial T*β*4 protein (8.49 ± 3.32%) (Figure 4) or the* E. coli*-derived 4×T*β*4 protein (18.12%; [24]).

### 3.5. Tobacco-Derived 4×T*β*4 Protein Promotes Healing Wound in Balb/c Mice (*In Vivo*)

**Figure 5:**
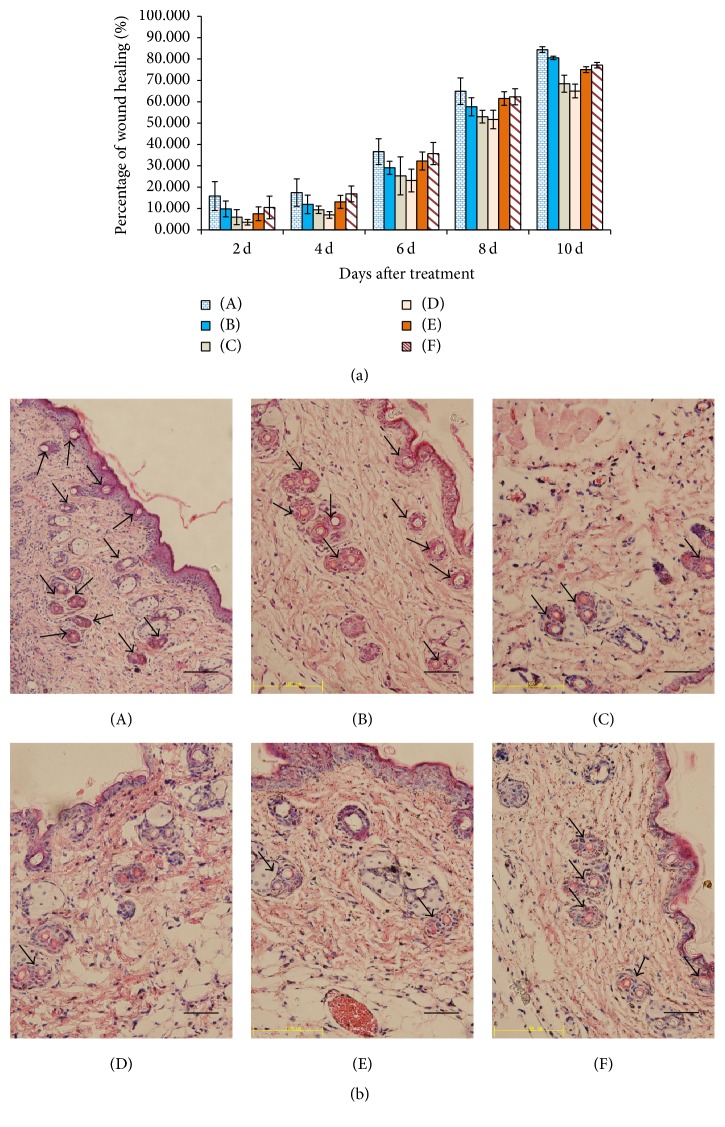
Tobacco-derived 4×T*β*4 promotes keratinocyte migration (a) and formation of blood vessels in the wound beds of mice (b). (A) Tobacco-derived 4×T*β*4 protein (5 *μ*g), (B) tobacco-derived 4×T*β*4 protein (2.5 *μ*g), (C) TSP (total soluble protein) (50 *μ*L) extracted from untransformed tobacco leaves, (D) 50 *μ*L PBS extraction buffer, (E)* E. coli-*derived 4×T*β*4 protein (5 *μ*g), and (F) commercially purchased standard T*β*4 (5 *μ*g). Black arrows indicate newly formed blood vessels and reepithelialization of the wound epidermis following topical treatments. Scale bars = 50 *μ*m.

The efficiency with which tobacco-derived 4×T*β*4 protein healed wounds and promoted keratinocyte migration was examined using a full thickness cutaneous mouse wound model. The lengths of the epidermal tongues from the wound edges were measured, and we observed that reepithelialization rates were higher in all of the treatment groups (transgenic tobacco-derived 4×T*β*4, commercial* Tβ4,* and recombinant* E. coli*-derived 4×T*β*4) than in the negative controls (nontransgenic tobacco crude protein and PBS buffer) and that the rate of reepithelialization sharply increased during days 6–8 after treatment. Moreover, on day 8, the rate of keratinocyte migration on the wound bed treated with transgenic tobacco-derived 4×T*β*4 (5 *μ*g) was the highest of all six treatments (Figure 5(a)). The reepithelialization rate in the transgenic tobacco-derived 4×T*β*4 (2.5 *μ*g) treatment group was slightly lower than both commercial T*β*4 and* E. coli*-derived 4×T*β*4 during days 2–8 after treatment. However, by day 10 the keratinocyte migration rate in tobacco-derived 4×T*β*4 (2.5 *μ*g) exceeded that of the positive controls (commercial T*β*4 and* E. coli*-derived 4×T*β*4) (Figure 5(a)).

Histological examination of tissue sections collected at day 8 after application revealed that tobacco-derived 4×T*β*4 protein promoted an increase in blood vessels in the wound bed. We observed that its angiogenic effects significantly exceeded those of both the positive (*E. coli*-derived 4×T*β*4 and commercial T*β*4 protein) and negative controls (nontransformed tobacco-derived crude protein and PBS) (Figure 5(b)).

## 4. Discussion

T*β*4, which has been described as the second most biologically active peptide in thymosin fraction 5, after thymosin *α*1 [1, 17], is a type of actin regulating protein that forms a complex with the actin monomer in a 1 : 1 ratio. This complexation prevents polymerization and so inhibits the formation of actin filaments. Actin monomers released from the T*β*4/actin complex, however, can drive polymerization reactions as a normal function of the cytoskeleton in cell scaffolding and motility [38, 39]. The sequence LKKTET of the 43-amino acid T*β*4 protein, which is strongly conserved between all *β*-thymosins, represents the “actin-binding motif” and is similar to the sequence of WH2 domains (Wasp Homology Domain 2, a name derived from the Wiskott-Aldrich syndrome protein) [40]. Previous research has suggested that T*β*4 may be useful for treating hard-to-heal wounds, including diabetic ulcers, bedsores and damaged corneas, and heart muscle injured by heart attacks and tumor biomarkers, as well as for curing various skin, central nervous system, and lung diseases [5, 10, 14, 17, 18, 21–23]. Consequently, there is great interest in the use of T*β*4 in clinical applications.

Genetically engineered T*β*4 proteins have been produced in prokaryotic expression systems, and* E. coli*-derived 4×T*β*4 proteins have been reported to promote wound healing* in vivo* [24]. However, high production costs and difficulties in extraction and purification of the protein still limit its practical application. Plant based expression systems provide an attractive alternative as they are typically less expensive and capable of yielding high protein expression levels, as well as providing a system in which protein folding and modification are more similar to equivalent processes in humans than in prokaryotes [22, 26–28, 34, 37]. In this study, we expressed recombinant 4×T*β*4 protein in transgenic tobacco lines and observed that it was more effective in healing wounds in Blab/c mice than were either commercial or* E. coli *derived T*β*4. Additionally, the tobacco-derived 4×T*β*4 protein was more effective at increasing the number of blood vessel counts in wound beds than were the other T*β*4 proteins tested (Figure 5(b)). Another prominent feature of the tobacco-derived 4×T*β*4 is that it can be applied directly to the wound, while 4×T*β*4 derived from* E. coli *expression systems requires extraction and purification prior to any clinical applications. We propose that plant-derived 4×T*β*4 may be more effective when treating acute injuries, such as burns, diabetic complications, bedsores, and corneal transplantations, than T*β*4 derived from other expression systems.

Much of the cost associated with plant expression systems comes from extraction and purification of the target protein, which is the major factor limiting the development of plant expression systems to produce therapeutic proteins. In the present study, we used plant-derived 4×T*β*4 to heal wounds in Blab/c mice via a direct application approach and showed that it was more efficient than both standard T*β*4 (commercial) and* E. coli*-derived 4×T*β*4 in promoting cell proliferation and wound healing in mice.

## 5. Conclusions

A 168 bp* Tβ4* gene was designed and synthesized according to tobacco codon usage, and a fused gene, comprising 4*×Tβ4* and a polyhistidine tag, was overexpressed in tobacco. Fourteen positive tobacco lines were obtained via* Agrobacterium-*mediated transformation. The successful expression of the T*β*4 protein in transgenic tobacco lines was confirmed by Western blot and ELISA analyses, and 4×T*β*4 protein concentrations as high as 3 *μ*g/g of fresh weight were detected in the transgenic tobacco leaves. The tobacco-derived 4×T*β*4 protein was more effective than either T*β*4 derived from* E. coli* or the chemically synthesized form at promoting splenic lymphocyte proliferation and wound healing when applied directly to the wounds of mice. This research lays the foundation for the development of therapeutic proteins using plant expression systems, particularly in the context of direct delivery administration methods.

## Supplementary Material

Appendix 1 presents the transformed and regenerating *Nicotianatabacum* at different developmental stages.
Appendix 2 showes the PCR results of the transgenic tobacco plants.
Appendix 3 appears 4×Tβ4 concentration of the young leaves indifferent transgenic tobacco lines.
Appendix 4 emerges expression situation of the recombinant 4×Tβ4 protein in transgenic tobacco leaves using Western blot.

## Abbreviations

BCIP/NBT: 5-Bromo-4-chloroindol-3-yl phosphate/nitro blue tetrazolium
ELISA: Enzyme-linked immunosorbent assay
MTT: 3-(4,5-Dimethylthiazol-2-yl)-2,5-diphenyl-2H-tetrazolium bromide
RPMI: Roswell park memorial institute
SDS-PAGE: Sodium dodecyl sulphate-polyacrylamide gel electrophoresis.
