# Supplementary figures and images for: A Tobacco-Derived Thymosin β4 Concatemer Promotes Cell Proliferation and Wound Healing in Mice
Source: Biomed Res Int. 2016 Jul 14;2016:1973413. doi: 10.1155/2016/1973413 (PMC4963596; doi:10.1155/2016/1973413)

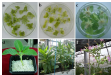

Supplement: Supplementary file 1 — Appendix 1 presents the transformed and regenerating Nicotianatabacum at different developmental stages. Appendix 2 showes the PCR results of the transgenic tobacco plants. Appendix 3 appears 4×Tβ4 concentration of the young leaves indifferent transgenic tobacco lines. Appendix 4 emerges expression situation of the recombinant 4×Tβ4 protein in transgenic tobacco leaves using Western blot. [file 1973413.f1.pdf]

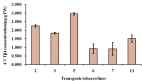

Supplement: Supplementary file 2 [file 1973413.f2.pdf]

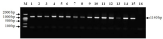

Supplement: Supplementary file 3 [file 1973413.f3.pdf]

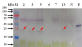

Supplement: Supplementary file 4 [file 1973413.f4.pdf]
