# Supplementary material for: A Tobacco-Derived Thymosin β4 Concatemer Promotes Cell Proliferation and Wound Healing in Mice
Source: Biomed Res Int. 2016 Jul 14;2016:1973413. doi: 10.1155/2016/1973413 (PMC4963596; doi:10.1155/2016/1973413)
Supplement: Supplementary file 5 [file 1973413.f5.pdf]

Supplementary Table 1 the recipe of RPMI 1640 medium*

Contents	Molecular weight	Concentration(mg/L)	mM
**Amino Acids**			
Glycine	75	10	0.133
L-Arginine	174	200	1.15
L-Asparagine	132	50	0.397
L-Aspartic Acid	133	20	0.15
L-Cystine 2HCl	313	65	0.208
L-Glutamic Acid	147	20	0.136
L-Glutamine	146	300	2.05
L-Histidine	155	15	0.0968
L-hydroxyproline	131	20	0.153
L-Isoleucine	131	50	0.382
L-Leucine	131	50	0.382
L-lysine hydrochloride	146	40	0.274
L-methionine	149	15	0.101
L-phenylalanine	165	15	0.0909
L-Proline	115	20	0.174
L-Serine	105	30	0.286
L-Threonine	119	20	0.168
L-Tryptophan	204	5	0.0245
L-Tyrosine disodium salt dehydrate	261	29	0.111
L-valine	117	20	0.171
**Vitamins**			
Biotin	244	0.2	0.00082
Choline chloride	140	3	0,0214
D-Calcium pantothenate	477	0.25	0.000524
Folic Acid	441	1	0.00227
Niacinamide	122	1	0.0082
Para-Aminobenzoic Acid	137	1	0.0073
Pyridoxine hydrochloride	206	1	0.00485
Riboflavin	376	0.2	0.000532
Thiamine hydrochloride	337	1	0.00297
Vitamin B12	1355	0.005	0.0000037
i-Inositol	180	35	0.194
**Inorganic Salts**			
Calcium nitrate(Ca)(NO3)2·4H2O	236	100	0.424
Magnesium Sulfate(MgSO4) anhydrous	120	48.84	0.407
Potassium Chloride(KCl)	75	400	5.33
Sodium Bicarbonate(NaHCO3)	84	2000	23.81

Sodium Chloride(NaCl)	58	6000	103.45
Sodium Phosphate dibasic (Na2HPO4) anhydrous	142	800	5.63
**Other Components**			
D-Glucose(Dextrose)	180	2000	11.11
Glutathione	30	1	0.00326

* From https://www.baidu.com/
